# A Novel System of Cytoskeletal Elements in the Human Pathogen *Helicobacter pylori*

**DOI:** 10.1371/journal.ppat.1000669

**Published:** 2009-11-20

**Authors:** Barbara Waidner, Mara Specht, Felix Dempwolff, Katharina Haeberer, Sarah Schaetzle, Volker Speth, Manfred Kist, Peter L. Graumann

**Affiliations:** 1 Department of Medical Microbiology and Hygiene, Institute of Medical Microbiology and Hygiene, University Hospital Freiburg, Freiburg, Germany; 2 Microbiology, Faculty of Biology, University of Freiburg, Freiburg, Germany; 3 Cell Biology, Faculty of Biology, University of Freiburg, Freiburg, Germany; Tufts University School of Medicine, United States of America

## Abstract

Pathogenicity of the human pathogen *Helicobacter pylori* relies upon its capacity to adapt to a hostile environment and to escape from the host response. Therefore, cell shape, motility, and pH homeostasis of these bacteria are specifically adapted to the gastric mucus. We have found that the helical shape of *H. pylori* depends on coiled coil rich proteins (Ccrp), which form extended filamentous structures *in vitro* and *in vivo*, and are differentially required for the maintenance of cell morphology. We have developed an *in vivo* localization system for this pathogen. Consistent with a cytoskeleton-like structure, Ccrp proteins localized in a regular punctuate and static pattern within *H. pylori* cells. Ccrp genes show a high degree of sequence variation, which could be the reason for the morphological diversity between *H. pylori* strains. In contrast to other bacteria, the actin-like MreB protein is dispensable for viability in *H. pylori*, and does not affect cell shape, but cell length and chromosome segregation. In addition, *mreB* mutant cells displayed significantly reduced urease activity, and thus compromise a major pathogenicity factor of *H. pylori*. Our findings reveal that Ccrp proteins, but not MreB, affect cell morphology, while both cytoskeletal components affect the development of pathogenicity factors and/or cell cycle progression.

## Author Summary

The human pathogen *Helicobacter pylori* lives in the hostile environment of the human stomach. *H. pylori* possesses a spiral shape and high motility that enable the bacterium to swim through the stomach lumen and to come into close contact with epithelial cells. High urease activity in the bacterium counterbalances the low pH within the stomach, in order to persist within the viscous mucus layer. In this work, we analysed the molecular basis of the spiral structure of *H. pylori*. We demonstrate that the helical cell shape depends on so called coiled coil rich proteins (Ccrp), which form extended filamentous structures *in vitro* and *in vivo*, and are differentially required for the maintenance of proper cell morphology. In most bacteria analysed so far, the actin-like protein MreB affects cell morphology. Contrarily, *H. pylori* MreB is not involved in the maintenance of cell shape, but affects the progression of the cell cycle. Mutant cells were highly elongated, characteristic for a delay in cell division, and contained non-segregated chromosomes. The persistence of *H. pylori* in the hostile environment of the human stomach depends on the activity of urease. Interestingly, *mreB* mutant cells displayed significantly reduced urease activity, revealing a novel connection between the cytoskeletal element and an enzyme, and thus with pathogenicity. These experiments show that *H. pylori* has a novel type of system setting up helical cell shape, which has not yet been described for any bacterium. Our work will allow studying *H. pylori* cell cycle and pathogenicity at a new visual level.

## Introduction

*Helicobacter pylori* is a Gram negative, highly motile, microaerophilic, spiral-shaped organism, which colonizes the stomachs of at least half of the world's population [1]. Infection of humans results in persistent gastritis, which can develop into peptic ulcer disease and adenocarcinoma [2],[3]. Motility is a key factor in the adaptation of infection, allowing for the penetration of the mucus and enabling the bacteria to colonize and to persist in the gastric lumen [4]. Both spiral shape and flagella contribute to the motility of this human pathogen. Whereas flagella of *H. pylori* have been studied intensively, our knowledge of the maintenance and establishment of spiral structure in *H. pylori* and in fact for any bacterium is marginal. In addition, nothing is known about any cytoskeletal protein in this pathogen.

Maintenance of cell morphology is highly important or essential for functioning and survival of most eukaryotic and prokaryotic cells. For many eukaryotic cells, it is also vital to be able to change the shape of the cell, and/or to be able to move via flexible extension/retraction of the cell membrane. Cytoskeletal elements actin and intermediate filaments are key elements of the eukaryotic cytoskeleton that controls cell morphology and cell rigidity. Due to its rapid polymerisation/depolymerization properties, actin is the driving force for motility involving membrane rearrangements, and is also involved in trafficking of vesicles and in cell division [5]. IF proteins, on the other hand, are characterized by extended coiled coil regions. The proteins are believed to be highly elongated and assemble into sheet structures based on extensive interactions between coiled coils [6]. IF like proteins provide mechanical strength to e.g. skin or blood vessel cells, and are involved in positioning of cellular organelles [7].

For most rod shaped bacteria analysed so far, the loss of genes affecting cell shape is lethal. *Escherichia coli* or *Bacillus subtilis* cells are unable to grow as round cells, into which they turn when gene products of *rodA*, *mreB*, or *mreC* are depleted. While RodA and MreC are membrane proteins, whose function is still unclear, MreB is an actin like protein that forms filaments *in vitro*, dependent on ATP [8]. *In vivo*, MreB forms filamentous helical structures underneath the cell membrane [9],[10]. In *B. subtilis* and in *Caulobacter crescentus*, these filaments are highly dynamic, and appear to move along the membrane with dynamics similar to those of eukaryotic actin [11],[12]. Movement of filaments is most likely based on ratchet-like extension of filaments at one end, and depolymerization (and thus shrinkage) at the other end. *E. coli* MreB and an MreB ortholog, Mbl, in *B. subtilis*, have been shown to interact with MreC [13],[14], which in turn appears to interact with enzymes that synthesize the extension of the murein sacculus [15]. Because the incorporation of new cell wall material occurs in a helical pattern [16], it has been proposed that the helical organization of MreB filaments in the cytosol may direct the helical localization of cell wall synthetic proteins within the periplasm/outside the cell. A disputed question is the effect MreB exerts on the segregation of duplicated chromosomes. Interfering with MreB levels or polymerization activity has been shown to strongly impair chromosome segregation in several organisms [10],[17], but arguments against a direct involvement of MreB in segregation have also been put forward [18].

The question of how bacterial cells can obtain a curved shape has only been investigated in the vibrio-shaped bacterium *C. crescentus*. *CreS* encodes for a coiled coil protein, crescentin, which has high similarity to IF proteins. Crescentin forms filamentous structures *in vitro* without the addition of any nucleotides. Deletion of *creS* leads to the generation of straight cells, and thus to loss of cell curvature, while the culture doubling time or any other obvious physiological aspect of the cell is not affected [19]. Crescentin localizes as a defined ribbon structure along the short side of the cells, suggesting that it forms a filamentous structure *in vivo* [19]. Recent evidence suggests that crescentin exerts its effect on cell curvature through mechanical control of cell growth [20].

In this work, we set out to analyse cytoskeletal elements in the human pathogen *Helicobacter pylori*. We have systematically inactivated genes encoding coiled coil-rich proteins, and for *mreB*. Surprisingly, deletion of *mreB* is not lethal, but affects a variety of cellular parameters, such as chromosome segregation, but not cell shape. Deletions of Ccrp (coiled coil rich proteins) genes have different effects on cell shape in different strains, from loss of helical shape to complete loss of a regular morphology. We have also established a system for the visualization of proteins in *H. pylori*, and show that Ccrp proteins have a specific pattern of localization, consistent with their function in cell shape maintenance.

## Results

### Genetic organization of genes predicted to encode for coiled coil rich proteins potentially serving as cell shape determinants

To gain insight into the question of how *H. pylori* gains its helical cell shape, we searched for elements similar to known cytoskeletal or cell morphological elements. Chromosomes of all *H. pylori* strains analysed contain a gene with high similarity to *mreB*, followed by a *mreC* gene. Like in *E. coli* and *B. subtilis*, the MreC gene product is predicted to contain a single membrane span, and coiled coil regions. No *mreD* gene could be found in the genomes, but a *rodA* like gene, and several *pbp* genes (not shown). Interestingly, all strains contain two genes that have already been suggested to encode for IF-like proteins (HP0059 and HP1143 in strain 26695) [19], which are predicted to contain several extended heptad repeat regions, but also a so-called stutter, where coiled coil 4 is clearly discontinued for few amino acids [21]. However, HP0059 is almost entirely composed of heptad repeat regions, and lacks the characteristic N- and C-terminal domains of IF proteins, which are predicted to be globular. According to their predicted secondary structure we suggest to term this class of proteins as “coiled coil rich proteins” (Ccrp). We designate the *H. pylori* HP0059 or HP1143 gene products as Ccrp59 or Ccrp1143, respectively.

Because of the genetic (and morphological) variability of *Helicobacter pylori*, we generated constructs in several different strains, to obtain information on the general validity of gene deletions or localization patterns of fusion proteins. We focussed our work on the reference strain 26695 (moderately motile), on KE88-3887, a hyper-motile variant of strain 26695, and on the clinical isolates G27 and 1061, all of which are relatively well amenable for genetic analysis.

### Deletion of HP0059 abolishes helical cell shape in several strains, while deletion of HP1143 affects cell morphology in strain 1061

**Figure 1:**
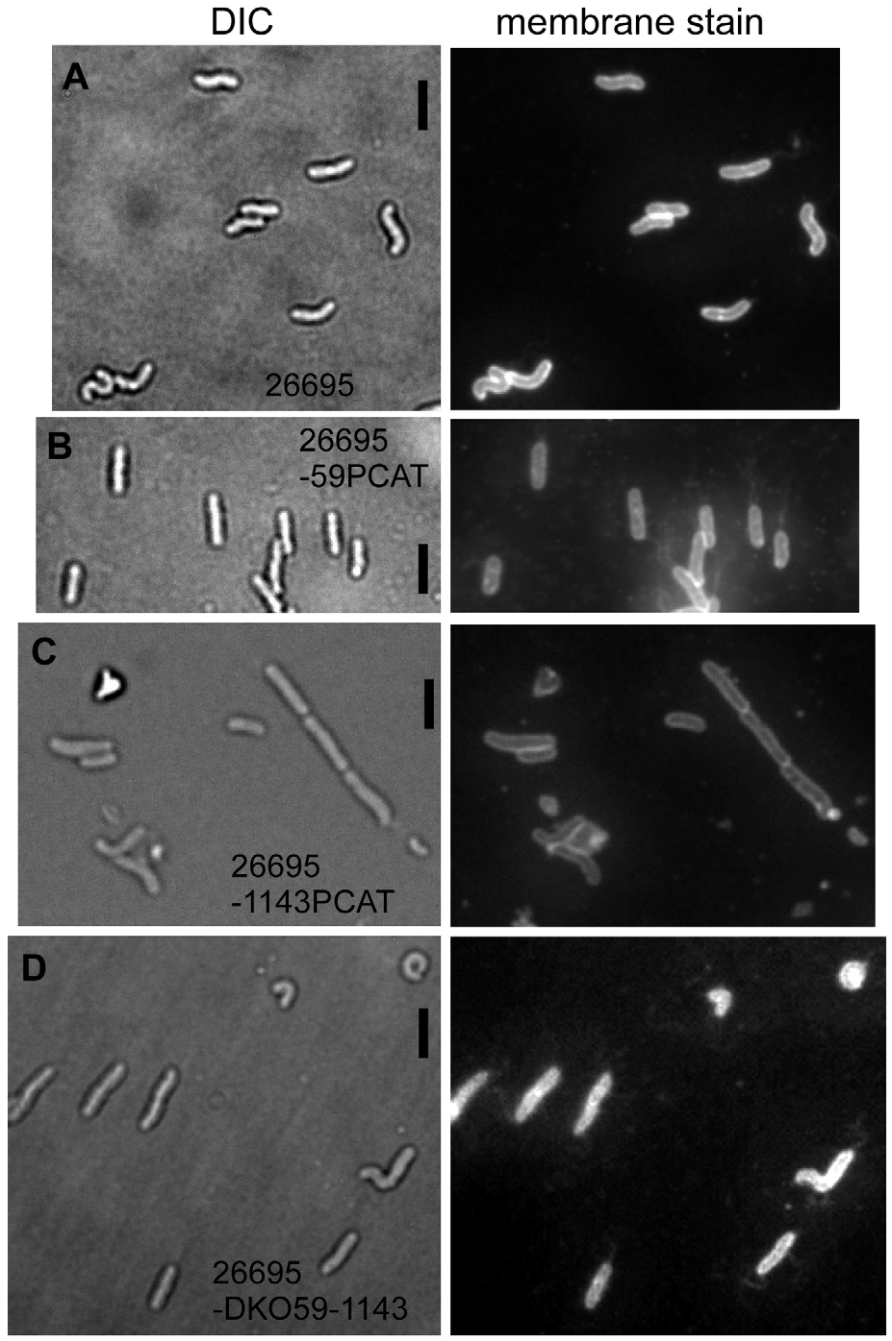
Deletion of genes encoding for Ccrp proteins in strain 26695. Images show fields of cells with Nomarski differential interference contrast (DIC) or with fluorescence of membrane stain. “pcat” indicates deletion of the corresponding gene, “DKO” refers to the double deletion of HP0059 and of HP1143. A) 26695, wild type strain; B) 26695-0059PCAT; C) 26695-1143PCAT; D) 26695-DKO; Black bars 2 µm.

**Figure 2:**
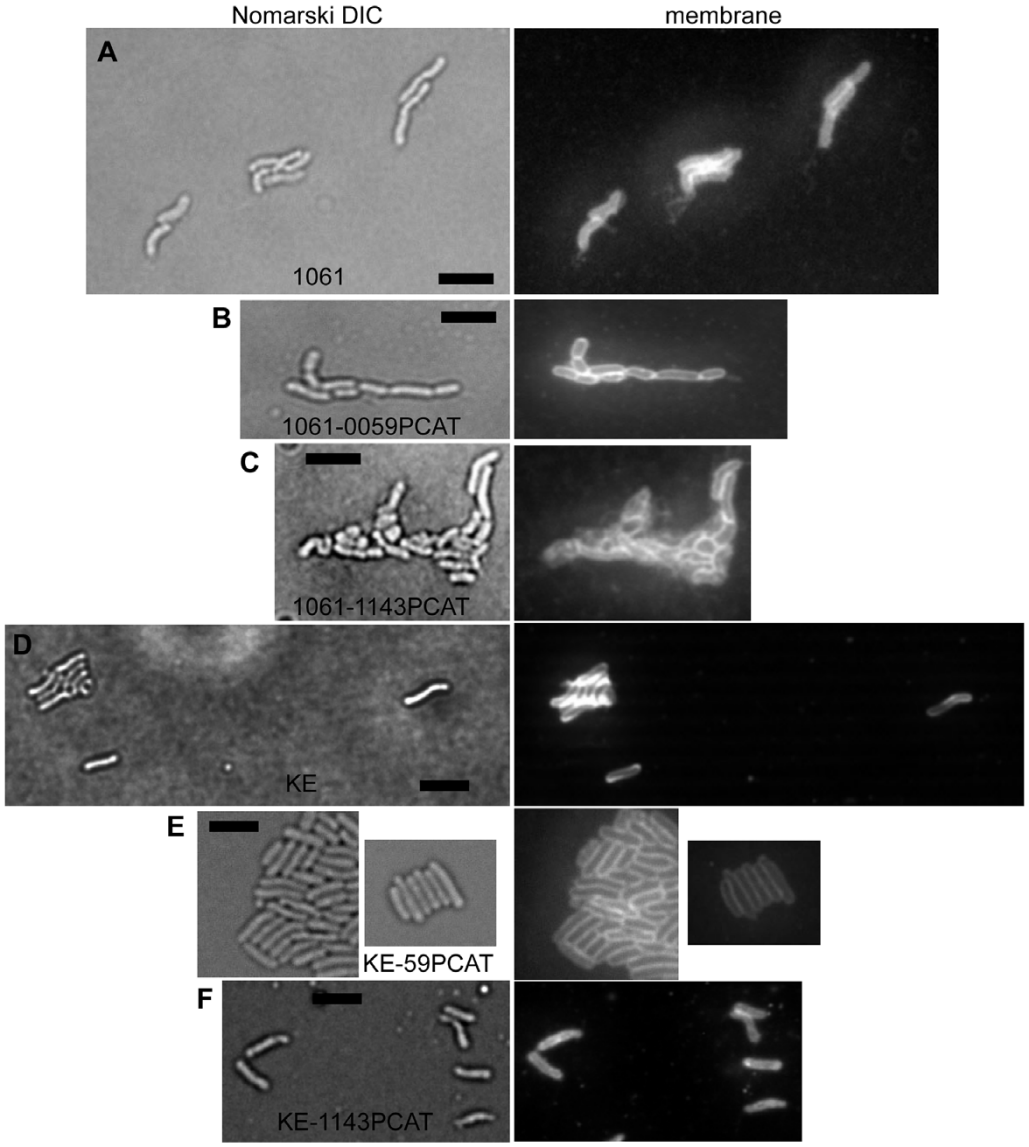
Deletion of genes encoding for Ccrp proteins in strain 1061 or KE88-3887. Images show field of cells with differential interference contrast (DIC) or with fluorescence of membrane stain. A) 1061, wild type strain; B) 1061-0059PCAT; C) 1061-1143PCAT; D) KE88-3887, wild type strain; E) KE-0059PCAT; F) KE-1143PCAT; Black bars 2 µm.

It should be noted that *H. pylori* strains have somewhat different morphologies. Strain 26695 is highly helical (Fig. 1A) (similar to KE88-3887, Fig. 2D), with an average of length of 3.0 +/− 0.5 µm (n = 72) and can be up to 4.0 µm in length, while cells of strain 1061 are much shorter with an average of length of 2.3 +/− 0.5 µm (n = 100) and their helical shape is less pronounced (compare Fig. 2A). Other strains of *H. pylori* also have varying degrees of cell curvature.

In order to study possible functions of genes predicted to encode for cell shape determinants, we inactivated genes HP0059 and HP1143 in *H. pylori* strains 26695, KE88-3887, G27 and 1061. To ensure expression of the downstream genes, all genes were disrupted by insertion of a *cat* gene driven by its own promoter but lacking a terminator. Growth analysis of all mutants revealed that inactivation of none of these genes showed any effect on the growth rate of *H. pylori*.

Interestingly, the inactivation of gene HP0059 resulted in the formation of 100% straight rods in strains 26695, KE88-3887 and G27 (Fig. 1B, Fig. 2E, and data not shown for G27), or 85% straight cells in strain 1061 (Fig. 2B), revealing a complete loss of the spiral shape in the absence of the HP0059 gene product. To rule out a possible downstream effect on gene HP0060, HP0060 was disrupted by introducing a *pcat* cassette. No change in growth or cell curvature could be detected compared with wild type cells (data not shown) showing that the loss of helical cell shape is due to the inactivation of gene HP0059. The same observation was made with strains 1061, KE88-3887 and G27 (data not shown). Inactivation of HP1143 had a mild effect on cell shape. Whereas 10 to 15% of 26695 wild type cells were straight (Fig. 1A), about 60% of HP1143 mutant cells were straight (Fig. 1C, or 52% for strain KE88-3887, Fig. 2F). These experiments show that the loss of genes HP0059 or of HP1143 affects cell curvature to different extents.

The deletion of HP1143 had an even more dramatic effect on cell shape in 1061 cells, about 70% of the cells were round, oval or irregularly shaped, while the remaining cells were straight or bulgy rod shaped (Fig. 2C). Single non-aggregated cells were basically undetectable. Thus, deletions of genes HP0059 and HP1143 have different effects on cell shape in different strain backgrounds. Absence of HP0059 generates loss of cell curvature in all strains tested, while lack of HP1143 results in a complete loss of cell shape in strain 1061.

*H. pylori* undergoes a transition from helical cells to coccoid cells upon prolonged starvation. We analysed whether *ccrp* mutant cells influence this morphological transition, whose mechanism is still poorly understood. Like wild type cells, all HP0059 or HP1143 mutant cells were coccoid 7 days after inocculation (i.e. 5 days into stationary phase), showing that the inactivation of Ccrp encoding genes does not influence the helical to coccoid transition.

### Deletion of both, HP0059 and of HP1143 exacerbates the cell shape defect in strain 26695

To investigate if HP0059 and HP1143 are genetically linked, we generated a strain from the parent 26695, in which both genes are deleted. Interestingly, cells of the double mutant strain displayed a variety of cell shapes: while 65% of the cells were straight, 30% had an irregular curved shape, and 5% had a highly bent shape, such that the cell ends came together (Fig. 1D), which is never observed for wild type cells. For strain KE88-3887, the double deletion resulted in even more highly bent cells (suppl. Fig. S1). These findings show that the loss of both Ccrp encoding genes leads to a complete loss of regular cell shape in strains 26695 and KE88-3887, and exacerbates the phenotype of the single gene deletions. The double deletion of HP0059 and HP1143 in strain 1061 was similar to that of the HP1143 single gene deletion, in that most (>80%) double mutant cells were round, oval or irregularly shaped, while the remaining cells were straight or bulgy rod shaped (data not shown).

### Ccrp59 forms filamentous structures *in vitro* and *in vivo*

**Figure 3:**
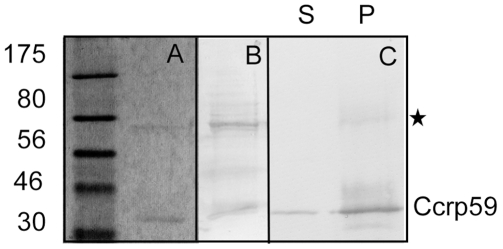
Spin down assays for Ccrp59-strep. A) Coomassie stained SDS PAGE showing purified Ccrp59-strep, the size of a monomer is indicated by the label. B) Western blot of purified Ccrp59-strep using streptavidin antibodies, showing that the upper putative dimer band consists of Ccrp59. C) Western blot of purified Ccrp59 of spin down assays of 20 µl fractions, S  =  supernatants, P  =  pellets resuspended in 20 µl SDS loading buffer; putative dimer band of Ccrp59 is marked with an asterisk.

**Figure 4:**
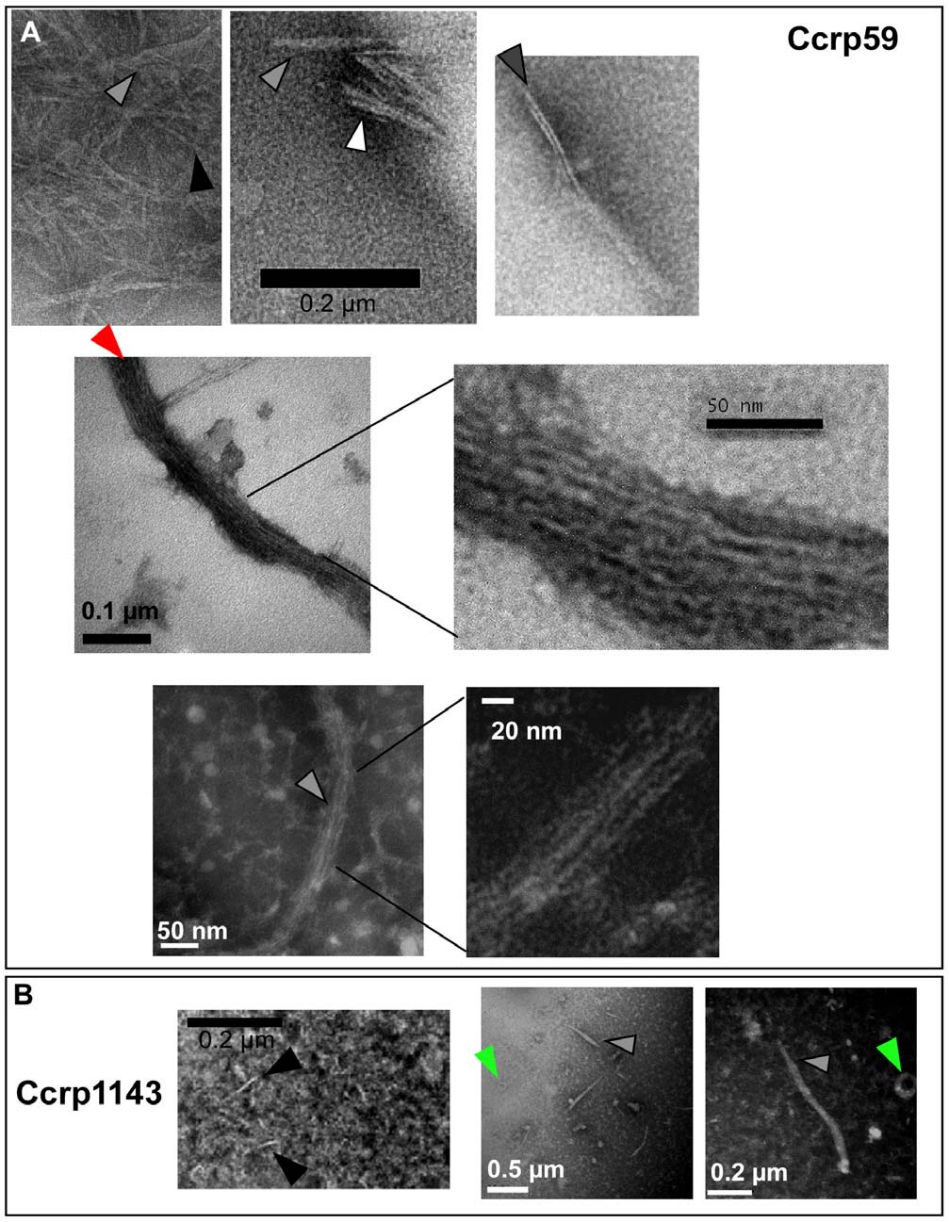
Electron microscopic images of purified Ccrp59 and Ccrp1143 proteins. A) Ccrp59; B) Ccrp1143; Black triangles indicate single filaments, grey triangles bundles of filaments, white triangles bundles of filaments that appear to split up into several individual filaments, hatched triangle a bundle of filaments having staggered ends, red triangle extended bundle of filaments and green triangles U-shaped structures in the case of Ccrp1143. All images from PTA stained samples, scale bars as indicated.

We wished to obtain insight into the biochemical properties of Ccrp proteins. Towards this end, we purified an N-terminally strep-tagged version of Ccrp59 to more than 95% purity (Fig. 3A). Ccrp59 could be purified in very low quantities as a soluble protein upon mild and short time (2 h) induction of the protein in *E. coli* cells, but appeared in inclusion bodies after prolonged induction. On SDS-PAGE, Ccrp59 migrated as monomer but also as a band that corresponded to a dimer (Fig. 3A), which is apparent in the Western blot in Fig. 3B. When subjected to centrifugation, a major proportion of Ccrp59 appeared in the pellet fraction (Fig. 3C), suggesting that it forms large assemblies. Over time (i.e. days to weeks), the amount of Ccrp59 in the pellet fraction increased (data not shown). When purified fractions were subjected to electron microscopy, it became clear that Ccrp59 forms extended filamentous structures *in vitro*, in the absence of any added cofactor (Fig. 4A), similar to IF-type proteins [21], and dissimilar to most other filament forming proteins. However, the fact that Ccrp59 can be purified as soluble protein clearly distinguishes it from IF proteins, which need to be refolded to be obtained in solution (compare Table 1 for dissimilarities and similarities). Filaments were generally straight, and present in bundles or sheets (Fig. 4A, grey triangles). The smallest observable filaments were 10 nm wide and about 50 nm long (Fig. 4A, black triangles); these may represent single Ccrp filaments. A closer investigation of the bundles showed that they consist of individual filaments (also 10 nm wide) that were positioned in parallel, and appeared to have staggered ends (hatched triangle). Bundles could frequently be observed to split into several single - or double filaments (Fig. 4A, white triangle), supporting the idea that Ccrp59 forms bundles or sheets of individual straight filaments. Most Ccrp59 bundles had a length of 120 to 160 nm, but bundles of more than 200 nm were also observed (Fig. 4A, red triangle). The diameter of individual filaments was in the range of 10 nm, similar to IF filaments from eukaryotic cells, while the larger filament bundles had a diameter of 30 to 60 nm. Interestingly, the length of Ccrp59 bundles as well as their width increased with time and with protein concentration. 5 to 7 days after purification, large bundles up to 950 nm length consisting of individual parallel filaments could be seen (Fig. 4A, lower panels), and were still observable after several weeks, showing that these structures are highly stable.

**Table 1:** Properties of IF and Ccrp proteins.

	Eukaryotic IF-protein	Ccrp59	Ccrp1143
Filamentous structures *in vitro*	+	+	+
Self assembly without cofactors	+	+	+
Filament diameter	10 nm	∼10 nm	∼10 nm
Bundles of filaments *in vitro*	No	+	No
pH sensitivity	+	n.d.	+
Structural sequence organization	central coiled coil domain flanked by amino- and carboxy-terminal domains of variable size and strructure	central coiled coil part but lacks non-coiled coil N – and C termini	long coiled coil region at its N terminus, which bears some features of IF proteins, but apparently lacks a stutter towards the end of the coiled coil region.
Recombinant protein	insoluble, need to be refolded	partially soluble	soluble

**Figure 5:**
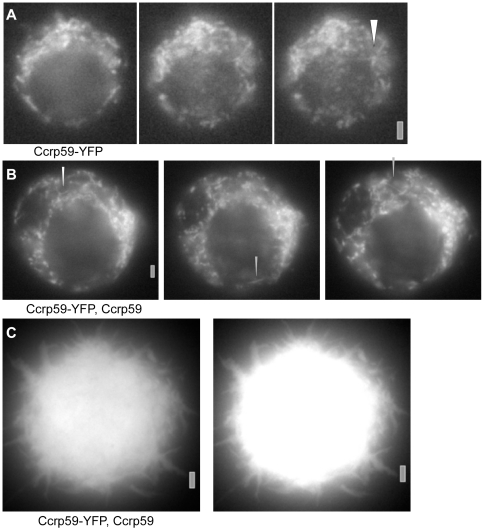
Expression of Ccrp59-YFP in S2 Schneider cells. A) 2 hours after induction of transcription, B) Co-expression of Ccrp59 and of Ccrp59-YFP 2 hours after induction of expression. White triangles indicate branched structures, grey triangle straight filament. The three panels show different Z section of the S2 cell (going from up to down). C) S2 cell 4 hours after induction of Ccrp59 and of Ccrp59-YFP. Right panel is identical to the left panel except for increased fluorescence intensity. Grey bars 2 µm.

Next, we wished to know if Ccrp59 can form filaments *in vivo* in a heterologous system. Accordingly, we transfected *D. melanogaster* S2 Schneider cells (derived from macrophages) with a Ccrp59-YFP fusion. Straight filamentous structures of 1.7 µm±0.3 (n = 9) length, which were frequently branched, could be seen soon after induction of transcription of the fusion (Fig. 5A), albeit in few cells. These observations show that the Ccrp59-GFP fusion can form filaments *in vivo*. Interestingly, upon coinduction of wild type Ccrp59, straight and branched filamentous structures were observed in more cells (Fig. 5B), which measured 2.45 µm±0.43 (n = 42) µm on average. At later time points after induction, large aggregates filled the transfected cells, which appeared to consist of large bundles of filaments (Fig. 5C). Importantly, Ccrp59-GFP filaments were of uniform fluorescence over their full length, and were much longer than those seen in the EM. These data show that Ccrp59 forms extended filamentous structures *in vitro* as well as *in vivo* in a heterologous system. The observations also suggest that the intracellular concentration of Ccrp59 must be kept at a certain level to avoid crowding of the cytosol by uncontrolled polymerisation of Ccrp59.

### Ccrp1143 forms filaments *in vitro*

In order to obtain information on Ccrp1143, the protein was purified as Strep-tagged versions analogous to Ccrp59, and was fully soluble (Table 1). When subjected to electron microscopy, Ccrp1143 was observed as single straight filaments of 50 to 60 nm length (Fig. 4B, left panel). Interestingly, a change of the purification condition from pH 8 to pH 7 resulted in the formation of structures, which were about 600 nm long and more than 20 nm wide, as well as of round or U-shaped structures of about 50 nm in diameter (Fig. 4B, right panels, green triangles). U-shaped structures were never observed for Ccrp59, suggesting that they are intrinsic to Ccrp1143. Therefore, similar to IF proteins [21], pH conditions considerably affect the ability of Ccrp1143 to form extended filaments (see Table 1). Like Ccrp59, Ccrp1143 filaments were still observed several weeks after purification, and thus highly stable structures.

These experiments show that both Ccrp proteins form filaments *in vitro*, which share several properties with IF proteins, but are dissimilar to IF type proteins because both can be purified as soluble proteins without the need for refolding.

### Localization of Ccrp59 in *H. pylori*

We wished to obtain insight into the pattern of localization of cytoskeletal elements in live *H. pylori* cells. We adapted a system for the generation of GFP fusions for *Bacillus subtilis* cells to *H. pylori*, which allowed integration of the fusions at the original locus within the chromosome. This strategy was successful with strain 1061, and in some cases also with 26695, which does not easily take up and integrate plasmid DNA (in contrast to linear DNA).

**Figure 6:**
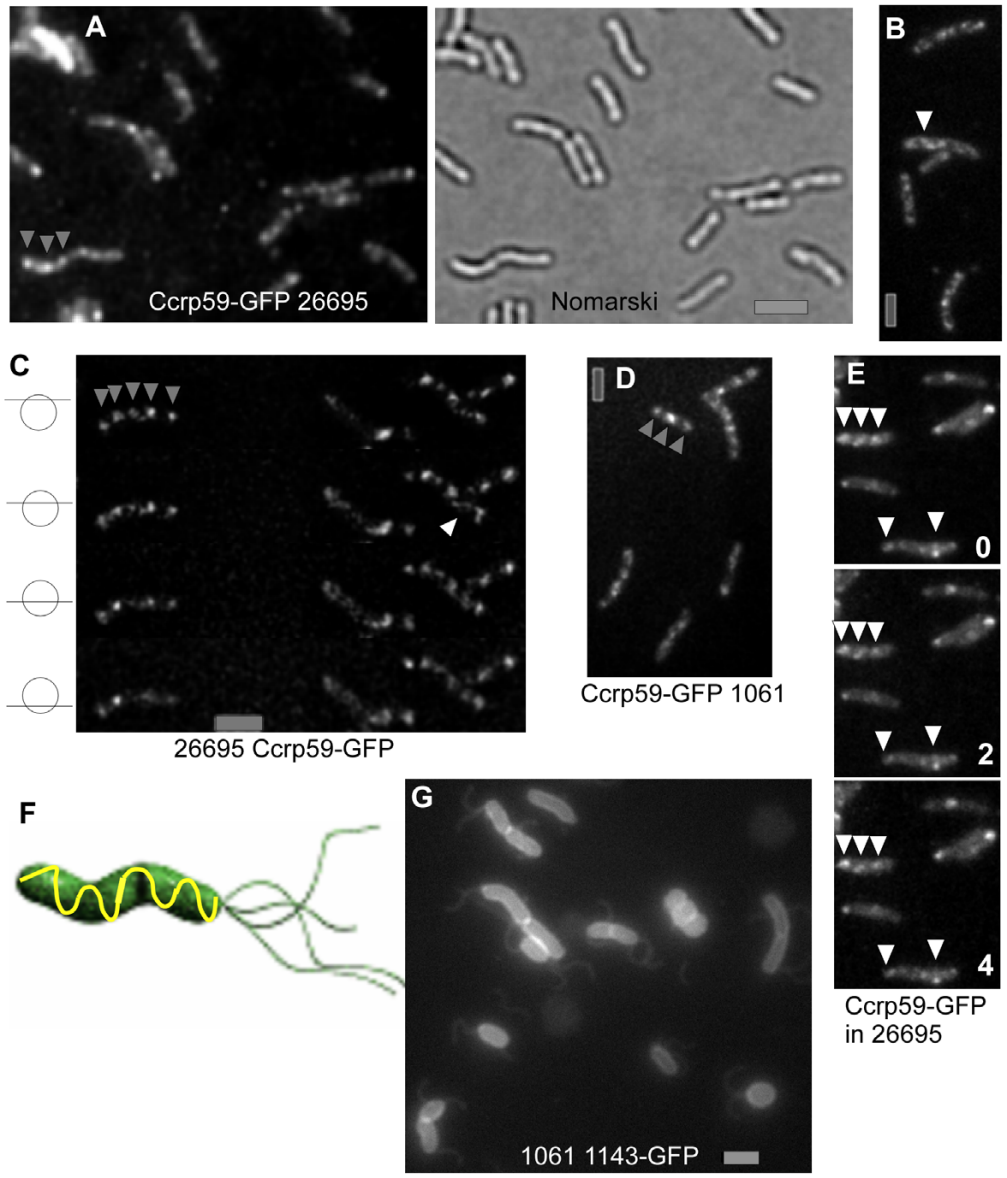
Localization of Ccrp proteins in different strains as indicated underneath the panels. A–E) Ccrp59-GFP. A) Cells expressing Ccrp59-GFP as sole source of the protein are helical like wild type cells, B–C) White triangles indicate possibly connected Ccrp59-GFP signals, C) 4 images taken in different planes as indicated, after 3D deconvolution, grey triangles indicate signals present in some planes and absent in others. D) Ccrp59-GFP in strain 1061, E) Time lapse with numbers indicating minutes between exposures, white triangles indicate distinct signals that do not move during the experiment, F) model for the possible localization pattern of Ccrp59 along the long axis of the cells. G) strain 1061 1143GFP expressing a fusion of Ccrp1143 with GFP. Note that the GFP fusion has a strong influence on cell shape. Grey bars 2 µm.

Ccrp59 was visualized through the generation of a C-terminal GFP fusion that was integrated at the original locus, such that it was expressed under the native promoter, and was the sole source of Ccrp59 within the cells. Cells of strain 26695 expressing Ccrp59-GFP were helical like wild type cells and not straight (Fig. 6A), showing that the fusion was functional, even in the absence of wild type Ccrp59. Discrete Ccrp59-GFP foci could be detected within exponentially growing cells (Fig. 6A–E). Small cells contained 2 to 3 foci, while the number of foci increased with cell size. Foci were not of uniform fluorescence, but showed different intensities. Foci with high fluorescence intensity (indicated by grey triangles in Fig. 6A, C and D) were positioned at relatively regular intervals within the cells, with an average of 0.89 µm±0.2 (n = 62) between the foci, and were frequently interspersed with foci of low intensity. Imaging of different Z-planes within cells and ensuing 3D deconvolution suggested that some of the foci were connected with each other (Fig. 6C). Due to the low cell diameter of H. pylori (0.78 µm), and because of the weak fluorescence of Ccrp59-GFP (which allows capturing of only 4–5 Z-planes) it was not possible to clearly determine if the foci are arranged in a helical pattern (which some images suggest), or in which other pattern. However, the data are compatible with a helical localization of Ccrp59 filaments along the long axis of the cells (Fig. 6F).

Ccrp59-GFP also localized in a very similar arrangement in strain 1061 (Fig. 6D), suggesting that the observed localization reflects the true positioning of Ccrp59 in several if not all H. pylori strains.

Importantly, time lapse microscopy showed that Ccrp59-GFP signals were not moving through the cells, but were stationary positioned over a period of 4 minutes (Fig. 6E). We did not observe any movement of Ccrp59-GFP foci in any of the 120 cells analysed. Thus, Ccrp59-GFP foci are not freely diffusing elements, supporting the idea that they may constitute cytoskeletal elements that are statically localized along the length of the cells.

We have not been able to generate a functional Ccrp1143-GFP fusion. We created a strain derived from 1061 in which a complete Ccrp1143-GFP fusion was integrated into the original locus by single crossover, such that the fusion as well as the wild type gene HP1143 were present within the *H. pylori* chromosome. Between 40 to 60% of these cells showed abnormal cell shape (Fig. 6G), and contained one to two distinct Ccrp1143-GFP foci at random places within the cell (data not shown), suggesting that the Ccrp1143-GFP fusion is dominant negative. These data reinforce the idea that a loss of function of Ccrp1143 leads to aberrant cell morphology.

### Ccrp encoding genes are heterogeneous in size and sequence between different *H. pylori* strains

As mentioned above, *H. pylori* strains can have different degrees of helical cell curvature and different cell lengths. Occasionally, laboratory strains lose cell curvature altogether and become rod shaped. To investigate if differences in genes encoding for Ccrp proteins may be the basis for this phenomenon, we amplified the gene region of HP0058 up to the beginning of gene HP0060 from different strains and sequenced the PCR products. Interestingly the whole region differs in length from about 1500 bp in strain 26695, 1600 bp in strain J99, about 1000 bp in strain 1061 up to only 550 bp in strain SS1 (mouse adapted). This is in agreement with a previous study that showed that HP0059 is among the most divergent genes in *H. pylori* [22]. Analysing HP0059 (encoding Ccrp59) itself, the size of 855 bp, 984 bp, 750 bp or 500 bp in strains 26695, J99, HPAG1 and 1061, respectively, was determined. Gene jhp0050 (is similar to HP0059) in strain J99 is 663 bp long. Because strain KE88-3887 is a hyper-motile variant of strain 26695 both strains contain the same HP0059 sequence.

### Deletion of *mreB* affects cell division and chromosome segregation, but not cell shape

**Figure 7:**
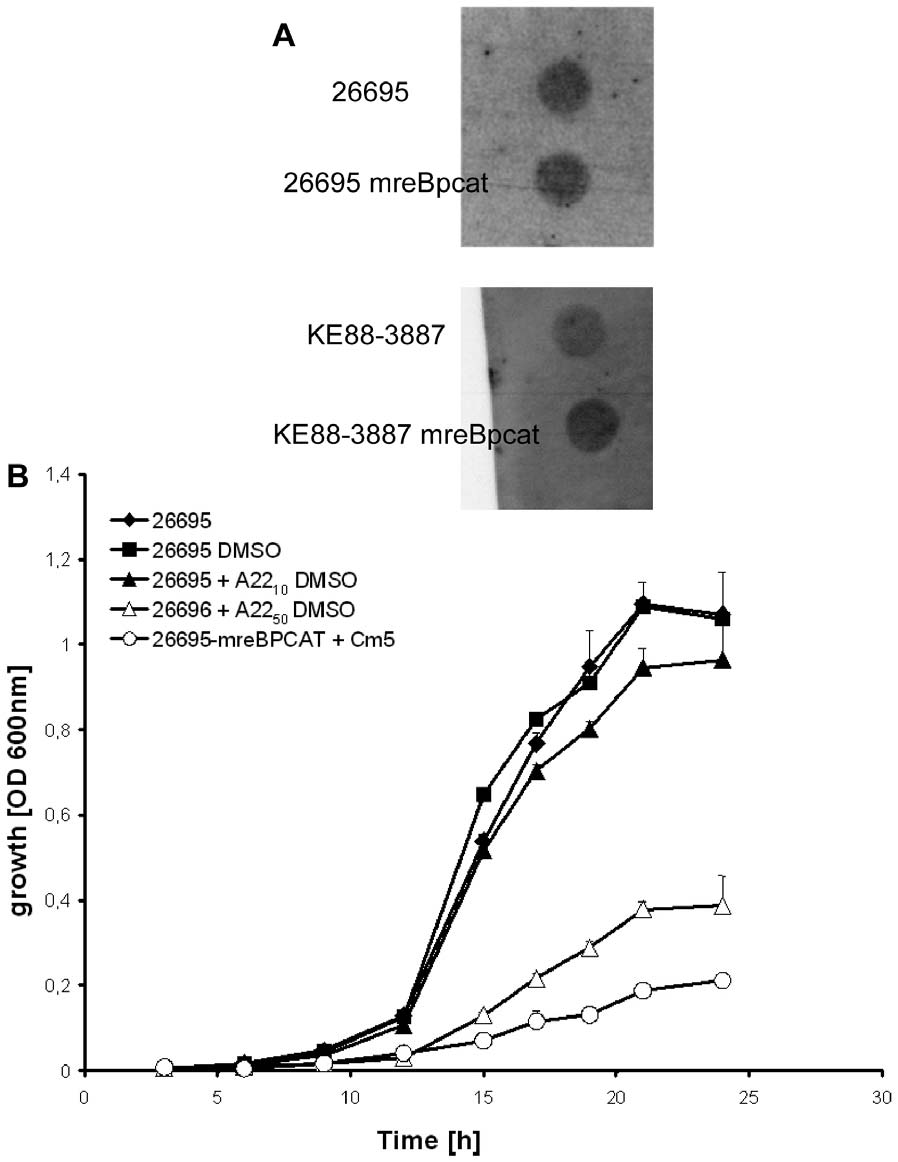
Influence of MreB on growth. A) Dot-blot hybridization of total RNA with probes specific for *mreC* mRNA. Upper dots show wild type cells, lower dots *mreB* deletion strains. The integrity of whole-cell mRNA was tested by agarose gel electrophoresis (data not shown). B) Growth of *H. pylori* strains in BBF broth, 26695 wild-type (rhombuses), wild-type after addition of the solvent DMSO (squares), wild type after addition of 10 µg/ml A22 (black triangles, note that these cells already show a defect in chromosome segregation) and 50 µg/ml A22 (open triangles), and the isogenic *mreB* mutant (circles) was determined by measuring the OD_600_. The data represent mean values from three independent determinations. Standard deviations are indicated.

To our surprise, it was possible to generate a deletion of the *mreB* gene, through a replacement of the gene with a chloramphenicol acetyltransferase cassette. Therefore we inactivated the *mreB* gene in three different strains indicating that this result was not strain dependent. The generated mutant cells were able to grow, albeit at strongly reduced growth rate compared with wild type cells. To rule out an effect on the downstream *mreC* gene, we isolated total RNA from *mreB* mutants from different *H. pylori* strains and performed dot-blot hybridization with probes specific for the *mreC* gene, showing that *mreC* is expressed in Δ*mreB* cells like in wild type cells (Fig. 7A), ruling out a polar effect of the disruption of *mreB*. *MreB* mutant cells were obtained at a similar frequency compared with many deletions of non essential genes generated in our laboratory [23], strongly arguing against the generation of secondary suppressor mutations.

The growth rate of *mreB* mutant cells was severely decreased compared with wild type cells for strain 26695 (Fig. 7B) and for KE88-3887 and 1061 (data not shown). The growth curves of all wild type strains showed a lag phase of about 8 hours and an exponential increase in cell density until at least 25 h, whereas all *mreB* mutants (i.e. in the 3 different strains) displayed a highly decreased growth rate.

**Figure 8:**
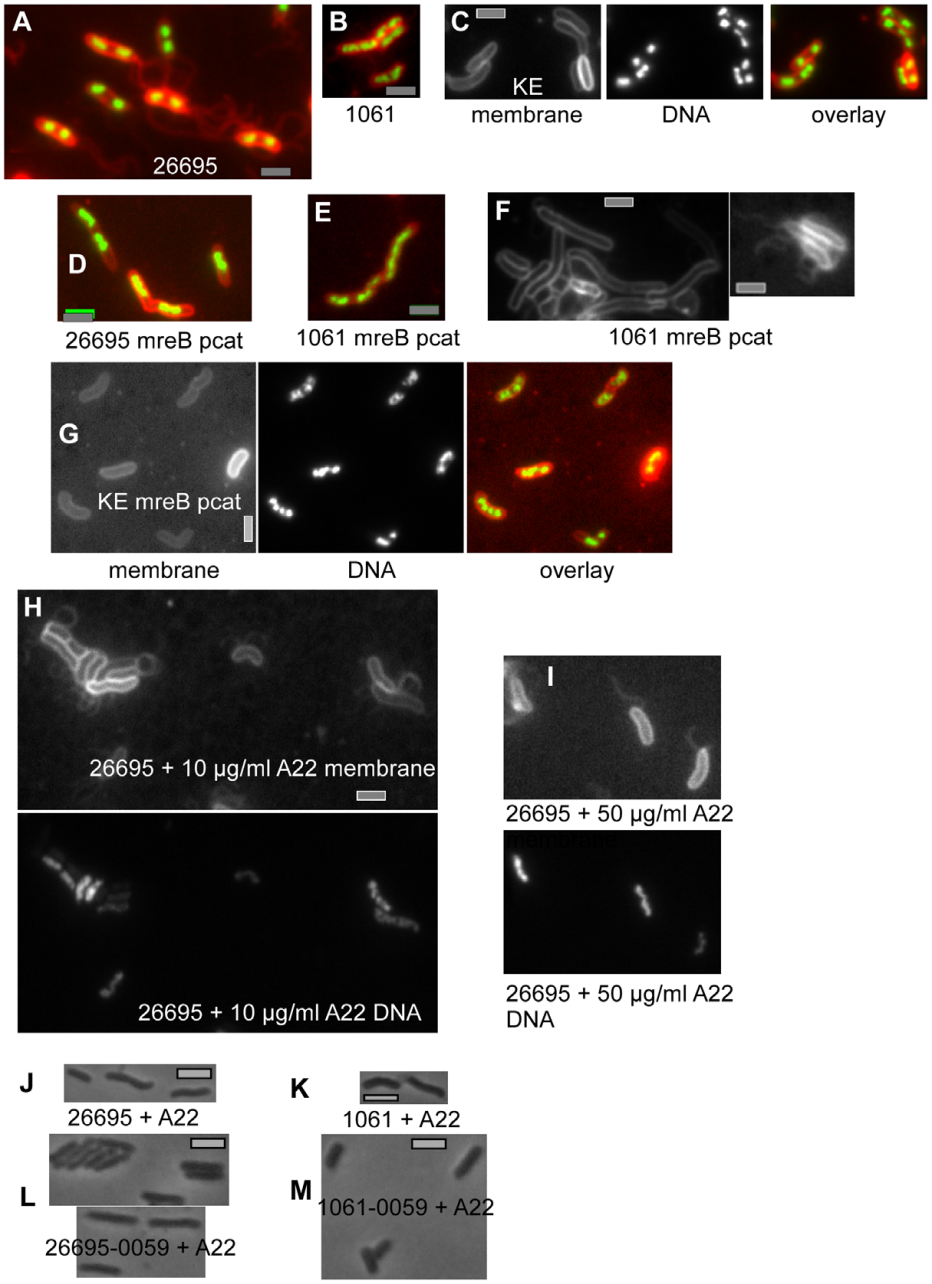
Deletion of *mreB* leads to a defect in chromosome segregation. A, B, D) overlays of DNA (green) and cell membrane (red). A–C) wild type cells of strains indicated on or underneath the image, D–G) *mreB* deletion strains as indicated at the images. E) membrane stain of *mreB* mutant cells showing cell elongation and presence of polar flagella (right panel). H–I) Membrane or DNA stain of cells 2 h after addition of A22, which inhibits MreB polymerization. J–M) Phase contrast images of cells 4 hours after addition of A22. J) 26695 wild type cells, K) 1061 wild type cells, L) 26695 HP0059 deletion cells, M) 1061 HP0059 deletion cells. Grey bars 2 µm.

Interestingly, there was no change in cell morphology of *mreB* mutant cells other than cell elongation in comparison with wild type cells (Fig. 8, compare A with D for 26695, B with E for 1061 and C with G for KE88-3887). *MreB* deleted cells were still helical and had the same average cell diameter of 0.78 µm (n>100 cells) than that of wild type cells. Cell elongation can be easily seen in Fig. 8F. Interestingly, *mreB* mutant cells showed a strong defect in the segregation of chromosomes. In contrast to wild type cells of all strain, which contained one, two, or (rarely) three well defined nucleoids (Fig. 8A, B and C), *mreB* mutant cells contained brightly staining bilobed nucleoids and large DNA free cell regions (Fig. 8D, E and G). Similar to *C. crescentus smc* mutant cells that have a strong segregation defect [24], no anucleate *mreB* mutant *H. pylori* cells were observed. Fluorescence intensity of the bilobed nucleoids in mutant cells was similar to that of separated nucleoids in large wild type cells, while the length of the bilobed nucleoids was twice of that of single segregated nucleoids in wild type cells, showing that the nucleoids in mutant cells contain two largely duplicated chromosomes demonstrating a separation delay. *MreB* mutant cells from strain 1061 frequently contained a single extended non-segregated nucleoid in spite of the large cell size (Fig. 8E), showing that loss of MreB strongly affects chromosome segregation in *H. pylori* cells. MreB mutant cells from strain 1061 could reach more than 3 times the normal cell size (Fig. 8F). Blocking of chromosome segregation leads to a delay in cell division in bacteria such as *E. coli* or *B. subtilis* [25], suggesting that most likely, the elongation of cells lacking MreB is due to the defect in chromosome partitioning.

To obtain further insight into the function of MreB, we treated *H. pylori* cells with A22, which was reported to mediate disassembly of MreB filaments *in vivo* [26]. Addition of a low amount of A22 (10 µg/ml) to exponentially growing *H. pylori* cells led to the formation of abnormally shaped nucleoids (Fig. 8H), and the addition of 50 µg/ml A22 resulted in a similar phenotype than the *mreB* deletion: only 2 doubling times after addition of A22, nucleoids no longer separated into two (Fig. 8I), and growth proceeded at an extremely slow rate (Fig. 7B, open triangles). Resuspension of cells after A22 treatment into fresh growth medium fully restored growth of cells, showing that interfering with MreB function transiently and rapidly leads to disturbance of the cell cycle, but does not kill the cells under experimental conditions. The fact that A22 treatment resulted in a phenotype that closely resembles that of an *mreB* deletion reinforces the idea that the phenotype is not masked by a secondary mutation but is due to the inactivation of MreB activity.

The lack of MreB did not interfere with the formation of polar flagella (Fig. 8F, right panel). Our data suggest that MreB plays a direct or indirect role in the progression of the cell cycle, but not in cell shape determination.

We also examined the possible link between Ccrps and MreB, because the effect of the lack of MreB on cell shape could potentially be masked by the presence of Ccrps. Therefore, we treated HP0059 mutant cells with A22, and visualized the effect of inhibition of MreB on cell shape. While wild type cells of strain 26695 (Fig. 8J) or of strain 1061 (Fig. 8K) remained helical during addition of A22, mutant cells of strain 26695 (Fig. 8L) or of strain 1061 (Fig. 8M) remained straight and rod shaped like the non-treated cells, and displayed the same degree of growth retardation as the wild type cells. These data support the findings that cell shape is not affected by the loss of MreB activity, but is determined by Ccrp proteins.

### Deletion of *mreB* leads to a decrease in urease activity

**Figure 9:**
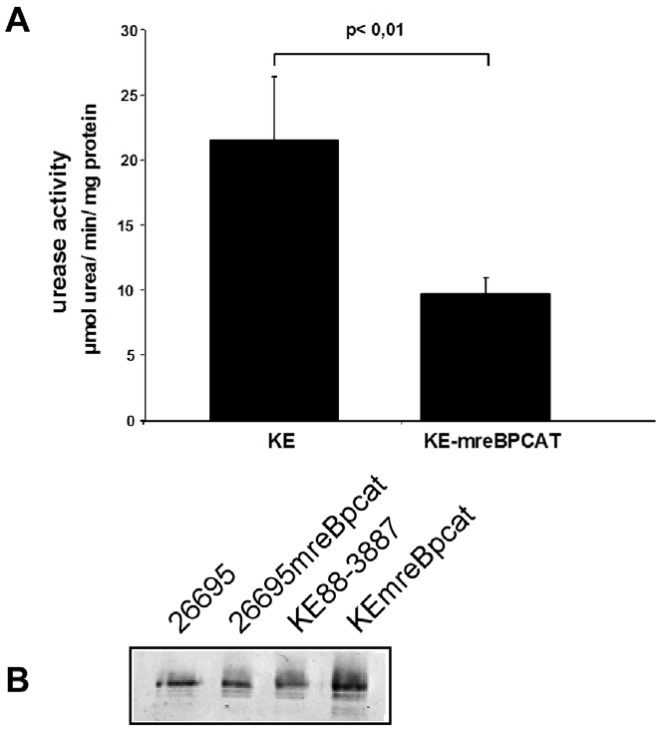
Influence of MreB on urease activity. A) Urease activity in strain KE88-3887 wild type cells and *mreB* mutant cells (KE mreBpcat). The bars represent mean values of urease activity obtained from four independent experiments. Standard deviations are indicated. B) Western blot using urease specific antiserum and strains as indicated above the lanes. Equal amounts of cells (and of protein) were loaded onto each lane.

The persistence of *Helicobacter pylori* in the hostile environment of the human stomach is ensured by the activity of urease. Urease catalyses the hydrolysis of urea into carbon dioxide and ammonia, which are buffering compounds essential to raises the pH in the microenvironment surrounding the cell [27] and to maintain the pH homeostasis in the bacterial cytoplasm [28]. Therefore, enzyme activity is essential for both early colonization events and for virulence [29],[30]. To test whether this major pathogenicity factor is affected by cytoskeletal elements, we determined urease activity in *mreB* mutant cells, and found a statistically significant (p<0.01) 2.5 fold decrease in activity in strain KE88-3887 (Fig. 9A), and a ∼6 fold decrease in strain 26695 (data not shown). Interestingly, addition of 1 µM NiCl_2_ restored urease activity up to wt level (data not shown). Western blot analysis showed that the urease level in the *mreB* mutant strain is similar to or even higher than that in the parental wild type strain (Fig. 9B). It is unclear how MreB exerts its effect on urease activity, but clearly, loss of this cytoskeletal element compromises *H. pylori* pathogenicity.

## Discussion

This report provides novel insight into the bacterial cytoskeleton and the function of cytoskeletal elements, and shows that the human pathogen *H. pylori* has a novel type of system for the establishment and maintenance of defined cell morphology. We show that two coiled coil rich proteins (Ccrp) are essential for the maintenance of proper cell shape in *H. pylori*, whereas the actin-like protein MreB is not involved in the generation of helical and/or rod cell morphology, like in many other bacteria. Deletion of gene HP0059 encoding for protein Ccrp59 resulted in the complete loss of helical cell curvature in strains 26695, KE88-3887, G27 and 1061. Loss of a second Ccrp protein, Ccrp1143, resulted in a mild reduction of cell curvature in strain 26695. However, in strain 1061, lack of Ccrp1143 resulted in a complete failure to maintain cell morphology, mutant cells were round or oval, or irregularly shaped. Thus, Ccrp proteins contribute differentially to cell morphology in different *H. pylori* strains, and are required for the maintenance of cell morphology in *H. pylori*. Intriguingly, in contrast to *mreB* and *ftsZ*, Ccrp encoding genes are highly variable both in terms of their length and in sequence between various *H. pylori* strains analysed in this study. It is thus plausible to propose that the great variety of cell shapes of *H. pylori* strains – from small bent cells to large and highly helical cells – stems from the nature of the Ccrp proteins. For example, Ccrp59 is much longer in strain 26695 than in 1061, which produces smaller and less helical cells than 26695. Thus, loss of Ccrp1143 in strain 26695 may be compensated for by Ccrp59, while Ccrp59 of strain 1061 may not be able to do so. Unfortunately, we do not have sophisticated genetic tools at present to test these intriguing ideas, and clearly, the situation is more complicated, because of the differential contribution of Ccrp proteins in different strains.

We show that both Ccrp proteins form extended filaments *in vitro*. Ccrp59 forms bundles of filaments *in vitro*, in the absence of any added cofactor, and is able to form extended filaments in macrophage cells, and thus in the absence of any cofactor from *H. pylori*. Ccrp59 bundles clearly consist of parallel stacks of filaments, which appear to be arranged in a staggered fashion, as is proposed to be the case for IF filaments from eukaryotic cells [6]. However, both Ccrp proteins identified in this study are initially soluble proteins when expressed in *E. coli* cells, and Ccrp59 lacks characteristic N and C terminal domains of IF proteins, showing that Ccrps are distinct from IF proteins. Possibly, Ccrp proteins are evolutionarily older versions of IF proteins, or even unrelated to IFs, and possibly a novel class of cytoskeletal elements in bacteria.

Towards a further analysis of Ccrps, we localized Ccrp59 in *H. pylori* cells. We found that a functional Ccrp59-GFP fusion forms distinct foci, whose position did not change over a time of several minutes, along the length of the cells. Thus, Ccrp59 is not a freely diffusing cytosolic protein, but remains at fixed positions, and may thus serve as a cytoskeletal structure that affects cell morphology. Due to the narrow width of *H. pylori* cells and the relatively weak fluorescence of Ccrp59-GFP, it was not possibly to unequivocally determine if the foci are arranged in a helical pattern. Because of the fact that Ccrp59 forms extended filaments, which can be longer than *H. pylori* cells, *in vitro*, and when expressed in S2 macrophages, we favour the idea that the foci consist of filaments that are connected and run along the long axis of the cells (Fig. 6F). Rigid Ccrp59 filaments could exert force onto the cell membrane and this way lead to a helical twist in the cell wall during synthesis. Alternatively, a helical growth pattern could also be achieved through a possible interaction of Ccrps and proteins that synthesize the cell wall, which would be positioned in a helical arrangement. MreB positions cell wall synthetic enzymes in *B. subtilis* [31], but *H. pylori* MreB clearly does not affect cell shape, so this function could be performed by Ccrps. Coiled coil rich/IF-like protein crescentin in *C. crescentus* forms a filamentous structure along the short axis of the cell, and likewise actin-like MamK and MamJ in magnetotactic bacteria, which align magnetosomes in a straight line along the short axis of the helical cells [32],[33]. On the other hand, the cytoskeletal element CfpA, found exclusively in spirochaetes, is part of filaments running along the long axis of the highly helical cells [34], which even persist and retain their helical path when the cells are gently lysed. Interestingly, CfpA is also predicted to contain a high degree of coiled coils. It will be important to determine the nature of the foci formed by Ccrp59-GFP within the cells, and to identify factors that interact with Ccrps in *H. pylori*, to find out how the proteins mediate the generation of helical curvature of the cells.

In *C. crescentus*, the IF-like protein crescentin is essential for the generation of cell curvature [19], while MreB is indispensable for the maintenance of rod shape, in striking contrast to *H. pylori*, where cell shape depends on two Ccrps, but not on MreB. Moreover, Ccrp59 is clearly different from crescentin, because crescentin forms individual long filaments [19], and not parallel bundles of filaments like Ccrp59. In *S. coelicolour*, the filament-forming coiled coil rich protein FilP affects cell rigidity, but not cell shape [35], while MreB is involved in differentiation (sporulation), but does not play a role during vegetative growth [36]. Our findings show that *H. pylori* employs a novel concept for the generation of complex cell shape and suggest that Ccrp proteins may set up complex cell shape in many other bacteria that contain MreB (which may serve different functions), and also in bacteria lacking *mreB*, such as Corynebacterium, which is rod shaped.

We also addressed the question of the function of MreB in *H. pylori*. *MreB* mutant *H. pylori* cells are viable, but grow much more slowly than wild type cells. Strikingly, mutant cells contained non-segregated but strongly fluorescent (and thus duplicated) chromosomes, and were highly elongated. Because a defect in chromosome segregation leads to a delay in cell division, cell elongation in *mreB* mutant cells most likely stems from the delay in cell cycle progression. Thus, in *H. pylori*, MreB affects chromosome segregation, but not cell shape, while in other bacteria, the observed defect in chromosome segregation may be due to an indirect effect caused by the loss of cell shape. Strikingly, *mreB* mutants contain considerably lower levels of urease activity, whereas the amount of urease is unchanged. At present, we have no clear indication as to how MreB might affect the activity of an enzyme. Possibly, MreB affects the activity of membrane proteins such as transporters, and the absence of MreB may thereby change intracellular levels of metals and ions. Indeed, a deletion of *mreB* in *B. subtilis* can be rescued by the addition of high concentrations of magnesium and sucrose [18], and urease activity in *H. pylori mreB* mutant cells can be rescued by an increase in the concentration of nickel, which is a limiting factor for the enzyme [37]. In any event, our findings severely alter the spectrum of cellular functions affects by MreB. Because high urease activity is a prerequisite for colonization and persistence of *Helicobacter pylori* in the hostile environment of the human stomach, we establish for the first time a connection, directly or indirectly, between the bacterial cytoskeleton and a pathogenicity factor.

To verify the different contributions of Ccrps and MreB in *H. pylori*, we added the MreB inhibitor A22 to HP0059 mutant cells. The addition of A22 resulted in slow growth in the mutant cells, which however retained their rod cell shape. Therefore, cytoskeletal elements in *H. pylori* strongly affect cell shape (Ccrps) and growth/pathogenicity (MreB), which emphasizes the potential to generate antibacterial chemicals by screening for compounds that affect the assembly of MreB and Ccrp proteins. The study of *H. pylori* at the level of cell biology and the investigation of its cytoskeleton has revealed a novel type of system for cell shape maintenance, and point to additional interesting features of its cell cycle that deserve further investigation.

## Materials and Methods

### Bacterial strains and growth conditions

Bacterial strains are listed in suppl. Table S1. *H. pylori* strains were routinely cultivated on Dent blood agar in a microaerobic atmosphere as described earlier [37]. Growth experiments were performed in Brucella broth with 5% fetal calf serum (BBF). Bacteria were precultured to an optical density at 600 nm (OD_600_) of approximately 1.0 in BBF and subsequently diluted 1∶150 in test media. Growth rate was assessed by optical density (OD_600_). All growth experiments were performed in triplicate and were repeated at least three times. *E. coli* strains were grown aerobically at 37°C in Luria-Bertani medium. When appropriate, growth media were supplemented with 50 µg/l ampicillin (Ap) or 20 µg/l chloramphenicol (Cm).

### DNA techniques and mutagenesis of *H. pylori*

Restriction and modifying enzymes (New England Biolabs, USA) were used according to the manufacturer's instructions. Cloning was performed in *E. coli* according to standard protocols. Plasmids were isolated with a QIAprep Spin Miniprep Kit from Qiagen (Qiagen 27104). The chloramphenicol-acetyl-transferase gene *cat_GC_* with (*Pcat*) promoter were amplified by PCR from plasmid pTnMax5 (suppl. Table S1) using primer CATS1 in combination with the primer CATAS1 (suppl. Table S2). The *Pcat* gene were fused to upstream and downstream DNA regions of mutagenized genes by using a modified version of the megaprimer PCR protocol [38],[39] as described earlier [23],[40],[41]. Marker exchange mutagenesis of *H. pylori* was performed by electroporation or natural transformation according to standard procedures [42]. *H. pylori* mutants carrying the *Pcat* gene inserted into the chromosome were selected by growth on Dent blood agar containing chloramphenicol (Cm) at concentrations of 20 mg/l. Correct insertion of *cat* and *Pcat* was verified by PCR analysis with appropriate primers listed in suppl. Table S2. All fluorescent tag vectors (see Protocol S1) were integrated into the *H. pylori* chromosome via single crossover integration, which was verified by PCR.

### Cell culture of Schneider cells and transient transfection

*D. melanogaster* S2 Schneider cells were grown in Schneider's *Drosophila* medium (Lonza Group Ltd.) supplemented with 5–10% fetal calf serum (FCS) at 25°C without addition of CO_2_. Cells were passaged every 2 to 3 days to maintain optimal growth. S2 cells were transfected using the cationic lipid Cellfectin (Invitrogen). The S2 cells were spread in a 6-well plate at 1×10^6^ per well in 3 ml medium with 5% FCS. Supercoiled plasmids (0.3 µg of each plasmid) were complexed with lipid (10 µl Cellfectin reagent) in 200 µl serum-free medium. The complex was incubated at room temperature for 15 min, filled up with serum-free medium to 1 ml and was added to cells from which the growth medium had been removed (cells were washed once with serum-free medium). After 18 hrs, the supernatant was removed and replaced by 3 ml of medium containing 5% FCS. After further incubation for 24 hrs, the production of the proteins was induced by adding CuSO_4_ to a final concentration of 1 mM.

### Production and analysis of recombinant proteins

Recombinant versions of the *H. pylori* HP0059 and HP1143 proteins were produced in *E. coli* using the StreptagTM protein expression system from IBA (Göttingen, Germany) according to the manufacturer's instructions (http://www.iba-go.de). The coding sequences from *H. pylori* strain 26695 were amplified using the primer pairs listed in Table S2 and cloned via the *Bsa*I restriction sites added as 5′-extensions (underlined) into plasmid pASKIBA-7TM (IBA-Göttingen). The plasmids were transferred to *E. coli* BL21 and expression was induced with 0.2 mg/l tetracycline. The bacteria were harvested by centrifugation and the recombinant proteins were purified to homogeneity on a Strep-Tactin^TM^ column according to the manufacturers' instructions. In case of HP1143 the coding sequence from *H. pylori* strain 26695 was amplified using the primer pairs listed in Table S2 with the Streptag sequence integrated and cloned via the *Nco*I and *Pst*I restriction sites into plasmid pETDuet-1 (Novagene). Protein expression was performed according to the manufacturer's instructions. For protein purification at pH 7, buffer W (100 mM Tris/HCl pH 8, 150 mM NaCl, 1 mM EDTA) as well as the buffer E (100 mM Tris/HCl pH 8, 150 mM NaCl, 1 mM EDTA, 2.5 mM Desbiotin) were adjusted to pH 7.

Spin down assays were performed as follows: 20 µl of purified protein fractions in buffer W (usually 24 h after elution, with storage at 4°C) were centrifuged at 13000 rpm in a bench centrifuge, after removal of the supernatant, the pellet was resuspended in 20 µl buffer W. SDS sample buffer were added and equal volumes of supernatant and pellet were subjected to SDS PAGE analysis.

### Analysis of urease activity

Urease activity was determined in fresh lysates by measuring ammonia production from hydrolysis of urea, as described previously [37],[43]. The concentration of ammonia in the samples was inferred from a standard NH_4_Cl concentration curve. Enzyme activity was expressed as µmol urea substrate hydrolysed min^−1^ (mg protein)^−1^.

### Electron microscopy analysis

Elution fractions of the Streptag purification and from gel filtration were applied to 200 mash copper grids and were negatively stained with 2% phosphotungstic acid pH 2.7 or with 1% uranyl acetate. Filaments were visualized under a Philipps/FEI CM10 (80000V) electron microscope equipped with a Bioscan Camera. Images were processed with Digital Micrograph (Gatan) software.

### Fluorescence microscopy

Fluorescence microscopy was performed on a Zeiss Axioimager microscope using a 100×Objective with A = 1.45. Cells were mounted on agarose gel pads on object slides. Images were acquired with a digital CoolSnap HQ CCD camera; signal intensities and cell length were measured using the *Metamorph 6.3 program* (Universal Imaging Corp., USA). 3D deconvolution was done using Autdeblur software. DNA was stained with 4′,6-diamidino- 2-phenylindole (DAPI; final concentration 0.2 ng/ml) and membranes were stained with FM4–64 (final concentration 1 nM). Filters used were: DAPI – ex360–370, dc400, em420–460, GFP – ex460–495, dc505, em510–550, FM4–64 ex480–550, dc570, em590. For acquisition of Z-stacks, 20 planes with a spacing of 0.2 µm were taken from bottom to top, or reverse. Signal intensities were measured in Metamorph program.

## Supporting Information

Protocol S1 Supplementary protocol
(0.04 MB DOC)

Figure S1 Phase contrast image of strain KE88-3887 DKO59-1143 (double HP0059 and HP1143 deletion strain).
(0.18 MB JPG)

Table S1 List of strains and plasmids
(0.09 MB DOC)

Table S2 List of oligonucleotides
(0.05 MB DOC)
