# Supplementary material for: A Novel System of Cytoskeletal Elements in the Human Pathogen Helicobacter pylori
Source: PLoS Pathog. 2009 Nov 20;5(11):e1000669. doi: 10.1371/journal.ppat.1000669 (PMC2776988; doi:10.1371/journal.ppat.1000669)
Supplement: Protocol S1 — Supplementary protocol (0.04 MB DOC) [file ppat.1000669.s001.doc]

**Supplementary protocol S1**

**Supplementary Material and Methods**

*RNA Hybridization*

Total RNA was extracted from 15 ml of *H. pylori* liquid culture grown to an optical density at 600 nm (OD600) of 1.0 using Trizol (Gibco) according to the manufacturer's instructions. RNA concentration and purity were determined by A260 and A280 measurements, and the quality of the preparation was assessed rapidly by electrophoresis in an agarose gel. RNA was denatured in RNA dilution buffer [1 × SSC (0.15 M NaCl plus 0.015 M sodium citrate), 50% formamide and 6.7% formaldehyde] at 68°C for 15 min and put on ice. RNA was applied to nylon membranes (Roche, Basel, Switzerland) using a Bio-Dot microfiltration apparatus (Bio-Rad, Hercules, CA). Following transfer, RNA was covalently bound to the membrane by cross-linking with 0.120 J/cm2 UV light of 254 nm wavelengths. Northern blot hybridization were performed according to a standard protocol described earlier [1]. PCR products carrying parts of the target genes were amplified with primers listed in suppl. table 3 and were used for the production of antisense RNA probes labeled with digoxigenin by in vitro transcription using T7 RNA polymerase (Roche) [2]. Northern hybridization and stringency washes were performed at 68°C, and bound probe was visualized with the DIG-Detection Kit (Roche) and the chemiluminescent substrate CPD-Star (Amersham Pharmacia, Roosendaal, NL).

*Generation of fluorescent protein fusions*

To create a C-terminal fusion of Ccrp59 and of Ccrp1143 with GFP (mut1), a 507 bp 3'-fragment or the entire 1544 bp gene were amplified by PCR using primer pairs 0059_up/0059_dw or HP1143gfp_up/HP1143gfp_dw, (suppl. table [2](http://www.pubmedcentral.nih.gov/articlerender.fcgi?artid=2216020&rendertype=table&id=T2)), respectively, and were cloned into *Apa*I and *Eco*RI restriction sites on pSG1164 vector [3]. All fluorescent tag vectors were integrated into the *H. pylori* chromosome via single crossover integration, which was verified by PCR.

To create a C-terminal fusion of Ccrp59 with YFP (eYFP) for transfection of the S2 cells, the coding sequence of HP0059 was amplified by PCR, using primer pair SS0059_up/SS0059_dw and was cloned into *Kpn*I and *Eco*RV restriction sites on pRmHa-3YFP containing eYFP [4]. Additionally, HP0059 was amplified by PCR using primer pair SS0059_up2/SS0059_dw2 and cloned into *Kpn*I and *Eco*RV restriction sites on pRmHa-3 [5].

*Western Blotting*

Serum raised against *H. pylori* urease B subunit (SE744 kindly provided by K. Melchers, ALTANA) was used for detection after gel electrophoresis (equal numbers of cells were applied, and equal amounts of protein were verified by Bradford tests) and blotting onto nitrocellulose membranes. Bound rabbit antibodies were detected with a protein A-alkaline-phosphatase-conjugate followed by incubation with Nitroblue-tetrazolium as substrate.

**References**

1. Bereswill S, Greiner S, van Vliet AH, Waidner B, Fassbinder F, et al. (2000) Regulation of ferritin-mediated cytoplasmic iron storage by the ferric uptake regulator homolog (Fur) of *Helicobacter pylori*. J Bacteriol 182: 5948-5953.

2. van Vliet AH, Kuipers EJ, Waidner B, Davies BJ, de Vries N, et al. (2001) Nickel-responsive induction of urease expression in *Helicobacter pylori* is mediated at the transcriptional level. Infect Immun 69: 4891-4897.

3. Lewis PJ, Marston AL (1999) GFP vectors for controlled expression and dual labelling of protein fusions in Bacillus subtilis. Gene 227: 101-110.

4. Kidane AG, Salacinski H, Tiwari A, Bruckdorfer KR, Seifalian AM (2004) Anticoagulant and antiplatelet agents: their clinical and device application(s) together with usages to engineer surfaces. Biomacromolecules 5: 798-813.

5. Savary-Bataille KC, Bunch SE, Spaulding KA, Jackson MW, Law JM, et al. (2003) Percutaneous ultrasound-guided cholecystocentesis in healthy cats. JVetInternMed 17: 298-303.
