# Supplementary figures and images for: A Novel System of Cytoskeletal Elements in the Human Pathogen Helicobacter pylori
Source: PLoS Pathog. 2009 Nov 20;5(11):e1000669. doi: 10.1371/journal.ppat.1000669 (PMC2776988; doi:10.1371/journal.ppat.1000669)

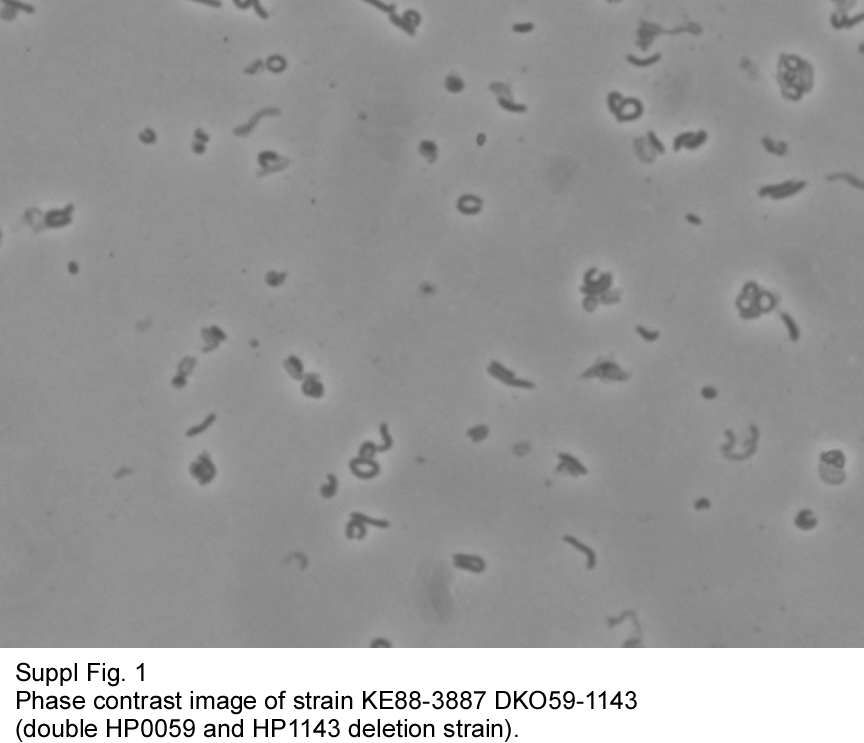

Supplement: Figure S1 — Phase contrast image of strain KE88-3887 DKO59-1143 (double HP0059 and HP1143 deletion strain). (0.18 MB JPG) [file ppat.1000669.s002.jpg]
