# Supplementary material for: A Novel System of Cytoskeletal Elements in the Human Pathogen Helicobacter pylori
Source: PLoS Pathog. 2009 Nov 20;5(11):e1000669. doi: 10.1371/journal.ppat.1000669 (PMC2776988; doi:10.1371/journal.ppat.1000669)
Supplement: Table S1 — List of strains and plasmids (0.09 MB DOC) [file ppat.1000669.s003.doc]

**Suppl. table S1**

|  |  |  |
| --- | --- | --- |
| **Strain or plasmid** | **Relevant characteristics** | **Source** |
|  |  |  |
|  |  |  |
| **Plasmids** |  |  |
| pTn*Max*5 | *lac*I^q^, *tnp*R, *tnp*A, *res*, *ori*_fd_, *cat*_GC_, Cm^R^ | [1] |
| pZERO-2 | Cloning vector, MCS in *lacZ'*, *neo*, Km^r^ | Invitrogen |
| pZERO-1 | Cloning vector, MCS in *lacZ'*, *zeo*, Zeo^r^ | Invitrogen |
| p0060PCAT | pZERO-2, Δ HP0058*::Pcat*, Cm^r^, Km^r^ | This study |
| p0059PCAT | pZERO-2, Δ HP0059*::Pcat*, Cm^r^, Km^r^ | This study |
| p1143-PCAT | pZERO-2, Δ HP1143*::Pcat*, Cm^r^, Km^r^ | This study |
| p1143-PNEO | pZERO-1, Δ HP1143*::Pneot*, Zeo^r^, Km^r^ | This study |
| pmreB-PCAT | pZERO-2, Δ *mreB::Pcat*, Cm^r^, Km^r^ | This study |
| pASK-IBA7 | Expression vector, *tetR*, *P_tet_*, *bla*, Ap^r^ | IBA, |
| pIBA7-0058 | pASK-IBA7 carrying the HP0058 coding sequence under the control of the *tet* promoter cloned in the *Bsa*I site | This study |
| pIBA7-0059 | pASK-IBA7 carrying the HP0059 coding sequence under the control of the *tet* promoter cloned in the *Bsa*I site | This study |
| pIBA7-1142 | pASK-IBA7 carrying the HP1142 coding sequence under the control of the *tet* promoter cloned in the *Bsa*I site | This study |
| pETDuet-1 | *bla* | Novagen |
| pETDuet-1143 | pETDuet-1 carrying the HP1143 coding sequence under the control of the T7promoter cloned between *NcoI* and *BamHI* site | This study |
| pSG1164 | *bla, cat, Pxyl-gfpmut1* | [2] |
| pSG1164-0059 | *bla, Pxyl-HP0059-gfpmut1, cat* | This study |
| pSG1164-1143 | *bla, Pxyl-HP1143-gfpmut1, cat* | This study |
| **Strains** |  |  |
| *E.coli* |  |  |
| BL21 | F^–^ *dcm ompT hsdS*(r_B_^–^ m_B_^–^) gal | Stratagene |
| DH5α | F^-^, φ80d*lacZ*ΔM15, Δ(*lacZYA-argF*)U169, *deoR*, *recA*1, *endA*1, *hsdR*17(rk^-^, mk^+^), *phoA*, *supE*44, λ^-^, *thi*-1, *gyrA*96, *relA*1 | Bethesda Research Laboratories |
| *H. pylori* |  |  |
| 26695 | wt, containing the entire cag PAI | [3] |
| 26695-60PCAT | 26695, ΔHP0060*::Pcat*, Cm^r^ | This study |
| 26695-59PCAT | 26695, ΔHP0059*::Pcat*, Cm^r^ | This study |
| 26695-1143PCAT | 26695, ΔHP1143*::Pcat*, Cm^r^ | This study |
| 26695-DKO-59-1143 | 26695, ΔHP0059*::Pcat*, Cm^r^, ΔHP1143*::Pneo*, Km^r^ | This study |
| 26695-mreBPCAT | 26695*, Δ mreB::Pcat*, Cm^r^ | This study |
| 26695-0059gfp | 26695, HP0059*-gfp* (at original locus) | This study |
| 1061-59PCAT | 1061, ΔHP0059*::Pcat*, Cm^r^ | This study |
| 1061-1143PCAT | 1061, ΔHP1143*::Pcat*, Cm^r^ | This study |
| 1061-mreBPCAT | 1061, Δ *mreB::Pcat*, Cm^r^ | This study |
| 1061-0059gfp | 1061, HP0059*-gfp* (at original locus) | This study |
| 1061-1143gfp | 1061, HP1143*-gfp* | This study |
| G27 | Clinical isolate | [4] |
| KE88-3887 | piglet-passaged strain 26695 | [5] |
| KE-59PCAT | KE88-3887, ΔHP0059*::Pcat*, Cm^r^ | This study |
| KE-1143PCAT | KE88-3887, ΔHP1143*::Pcat*, Cm^r^ | This study |
| KE-mreBPCAT | KE88-3887, Δ *mreB::Pcat*, Cm^r^ | This study |
|  |  |  |
|  |  |  |

**References**

1. Kahrs AF, Odenbreit S, Schmitt W, Heuermann D, Meyer TF, et al. (1995) An improved TnMax mini-transposon system suitable for sequencing, shuttle mutagenesis and gene fusions. Gene 167: 53-57.

2. Lewis PJ, Marston AL (1999) GFP vectors for controlled expression and dual labelling of protein fusions in Bacillus subtilis. Gene 227: 101-110.

3. Tomb JF, White O, Kerlavage AR, Clayton RA, Sutton GG, et al. (1997) The complete genome sequence of the gastric pathogen *Helicobacter pylori*. Nature 388: 539-547.

4. Xiang Z, Censini S, Bayeli PF, Telford JL, Figura N, et al. (1995) Analysis of expression of CagA and VacA virulence factors in 43 strains of *Helicobacter pylori* reveals that clinical isolates can be divided into two major types and that CagA is not necessary for expression of the vacuolating cytotoxin. Infect Immun 63: 94-98.

5. Hoffman PS, Vats N, Hutchison D, Butler J, Chisholm K, et al. (2003) Development of an interleukin-12-deficient mouse model that is permissive for colonization by a motile KE26695 strain of Helicobacter pylori. InfectImmun 71: 2534-2541.
