# Supplementary material for: A Novel System of Cytoskeletal Elements in the Human Pathogen Helicobacter pylori
Source: PLoS Pathog. 2009 Nov 20;5(11):e1000669. doi: 10.1371/journal.ppat.1000669 (PMC2776988; doi:10.1371/journal.ppat.1000669)
Supplement: Table S2 — List of oligonucleotides (0.05 MB DOC) [file ppat.1000669.s004.doc]

**Suppl. table S2.** Oligonucleotides used in this study

| **Gene^a^** | **Application** | **Primer** | **Sequence (5'→3')** | **Primer** | **Sequence (5'→3') ^b^** |
| --- | --- | --- | --- | --- | --- |
| HP0059 | Mutagenesis | 0058-L1 | AGCGAGCCAAGAATTAAGCG | 0059PCAT-R1 | 1- CGCGATAACTTGGTGGTTGA |
|  | Mutagenesis | CAT-0059-L1 | 2- AGCGCGATAGAGGATCTCAA | 0060-R1 | TTCTATCTCTTCTCCGCCGA |
| HP0060 | Mutagenesis | 0060-L1 | GCTAAGAGTGCTTCATGCGA | 0060PCAT-R1 | 1- GCGCCACTTGTATATCGTTC |
|  | Mutagenesis | CAT0060-L1 | 2- GACAAGATAGATGACGGCGT | 0060-R2 | ACTGCACACCTTCACATCCA |
| HP1143 | Mutagenesis | 1144-L1 | TGCCGAATAATGGAATTGAG | PCAT-1143-R1 | 1- AATTGGCTCTTGACACTTGG |
|  | Mutagenesis | CAT-1143-L1 | 2- TTGAGCAATTGAGAGCGGAT | 1142-R1 | CTCTCATTGTTATACCATGC |
|  | Mutagenesis | NEO-1143-L1 | CCTAGATTTAGATGTCT TTGAGCAATTGAGAGCGGAT | PNEO-1143-R1 | CGTACCGGTTCCAATTTT AATTGGCTCTTGACACTTGG |
| *mreB* | Mutagenesis | 1374-L1 | AGCACGCTAGATAGCATCAG | PCAT-mreB-R1 | 1- ATCGTGTTAGCCGTGCCTAA |
|  | Mutagenesis | CAT-mreB-L1 | 2- CACAGGAGAAGCCATACAAG | mreC-R1 | TCATCACGCTATAAGCGCAC |
| HP0059 | Expression | pASK7-0059-L1 | ATGGTAGGTCTCAGCGCATGGGAACATTCATTGAAAAATGTTTT | pASK7-0059-R1 | ATGGTAGGTCTCATATCATGGTTTTGGTTGTTTTGAGGGTTG |
| HP1143 | Expression | pETD-1143_up | tatccatggatgcaagaaaatcaaacccgtc | pERD-1143_dw | tatggatccctatttttcgaactgcgggtggctccaggggggatcttctaaatcctccccatc |
| HP0059 | Gfp-fusion | 0059_up | tatgggcccgctaactaacaagatcaccg | 0059_dw | ttcgaattcccctcgacctggttttggttgttttgaggg |
|  |  |  |  |  |  |
| HP0059 | Gfp-fusion | SS0059_up | TCAGGTACCATGGGAACATTCATTGAAAAATG | SS0059_dw | TCAGATATCCCCGCCTTATGGTTTTGGTTGTTTTGAGG |
|  | Gfp-fusion | SS0059_up2 | TCAGGTACCATGGGAACATTCATTGAAAAATG | SS0059_dw2 | TCAGATATCCCCGCCTGGTTTTGGTTGTTTTGAGGG |
| HP1143 | Gfp-fusion | HP1143gfp_up | tctgggcccgtatggttctctaaaacttg | HP1143gfp_dw | caagaattccccacctcctctcaaagcacaccacaaaaac |
|  | Gfp-fusion | 966 | CTAGAATTCCCCTCCACCGCCTTCACTAAAACCCACACGGC | 967 | TCAGGGCCCGGCACGACTGAAATTGGCG |
| *mreC* | Dot-Blot | S-mreC-L1 | GTTCTTCTTATATCAGCGAC | S-mreC-T7R1 | T7-CACGACTAATTCTTGCTTGA |
| *Pcat* | *cat* gene with promoter | CATS1 | TCCGGTTTTTGTTAATCCGCC | CATAS1 | TTACGCCCCGCCCTGCCA |
| *Pneo* | *neo* gene with promotor | KMS1 | AAAATTGGAACCGGTACG | KMAS1 | AGACATCTAAATCTAGG |
|  |  |  |  |  |  |

Gene numbers refer to the *H. pylori* 26695 genome sequence. The 5'-extensions used for fusion of PCR products to the *cat* gene by megaprimer PCR are labeled as follows:

1, (5'-GGCGGATTAACAAAAACCGGA), complementary to the 5'-region of the *cat* gene with promoter;

2, (5'-TGGCAGGGCGGGGCGTAA), complementary to the 3'-end of the *cat* gene;

T7, (5'-CTAATACGACTCACTATAGGGAGA) adds a T7 promoter sequence for creation of Dig-labeled antisense RNA.
